# Molecular Mechanisms of Proteins — Targets for SARS-CoV-2 (Review)

**DOI:** 10.17691/stm2020.12.6.11

**Published:** 2020-12-28

**Authors:** A.V. Morgun, V.V. Salmin, E.B. Boytsova, O.L. Lopatina, A.B. Salmina

**Affiliations:** Associate Professor, Department of Pediatrics; Krasnoyarsk State Medical University named after Prof. V.F. Voino-Yasenetsky, 1 Partizana Zheleznyaka St., Krasnoyarsk, 660022, Russia;; Professor, Head of the Department of Medical and Biological Physics; Krasnoyarsk State Medical University named after Prof. V.F. Voino-Yasenetsky, 1 Partizana Zheleznyaka St., Krasnoyarsk, 660022, Russia;; Infectious Disease Physician; Regional Clinical Hospital, 3a Partizana Zheleznyaka St., Krasnoyarsk, 660022, Russia; Professor, Department of Biological, Medical, Pharmaceutical and Toxicological Chemistry; Krasnoyarsk State Medical University named after Prof. V.F. Voino-Yasenetsky, 1 Partizana Zheleznyaka St., Krasnoyarsk, 660022, Russia;; Professor, Head of the Department of Biological, Medical, Pharmaceutical and Toxicological Chemistry Krasnoyarsk State Medical University named after Prof. V.F. Voino-Yasenetsky, 1 Partizana Zheleznyaka St., Krasnoyarsk, 660022, Russia;

**Keywords:** coronavirus infection, SARS-CoV-2, brain damage in COVID-19, blood-brain barrier, neuroinflammation, ACE2, CD147

## Abstract

The rapidly accumulating information about the new coronavirus infection and the ambiguous results obtained by various authors necessitate further research aiming at prevention and treatment of this disease. At the moment, there is convincing evidence that the pathogen affects not only the respiratory but also the central nervous system (CNS).

**The aim of the study** is to provide an insight into the molecular mechanisms underlying the damage to the CNS caused by the new coronavirus SARS-CoV-2.

**Results.:**

By analyzing the literature, we provide evidence that the brain is targeted by this virus. SARS-CoV-2 enters the body with the help of the target proteins: angiotensin-converting enzyme 2 (ACE2) and associated serine protease TMPRSS2 of the nasal epithelium. Brain damage develops before the onset of pulmonary symptoms. The virus spreads through the brain tissue into the piriform cortex, basal ganglia, midbrain, and hypothalamus. Later, the substantia nigra of the midbrain, amygdala, hippocampus, and cerebellum become affected. Massive death of neurons, astrogliosis and activation of microglia develop at the next stage of the disease. By day 4, an excessive production of proinflammatory cytokines in the brain, local neuroinflammation, breakdown of the blood-brain barrier, and impaired neuroplasticity are detected. These changes imply the involvement of a vascular component driven by excessive activity of matrix metalloproteinases, mediated by CD147. The main players in the pathogenesis of COVID-19 in the brain are products of angiotensin II (AT II) metabolism, largely angiotensin 1-7 (AT 1-7) and angiotensin IV (AT IV). There are conflicting data regarding their role in damage to the CNS in various diseases, including the coronavirus infection.

The second participant in the pathogenesis of brain damage in COVID-19 is CD147 — the inducer of extracellular matrix metalloproteinases. This molecule is expressed on the endothelial cells of cerebral microvessels, as well as on leukocytes present in the brain during neuroinflammation. The CD147 molecule plays a significant role in maintaining the structural and functional integrity of the blood-brain barrier by controlling the basal membrane permeability and by mediating the astrocyte-endothelial interactions. Via the above mechanisms, an exposure to SARS-CoV-2 leads to direct damage to the neurovascular unit of the brain.

## Introduction

Clinical and experimental results accumulated over the past few months indicate that the brain acts as a target organ for the COVID-19 pathogen [1, 2]. This virus called SARS-CoV-2 is known to enter tissues and organs by binding to the angiotensin-converting enzyme (ACE2), the associated serine protease (TMPRSS2) [3], and the extracellular matrix metalloproteinases inducer (CD147/EMMPRIN) [4–6]. Although main complications of COVID-19 are associated with lung tissue damage and impaired oxygen blood transport, an increasing number of clinical observations focus on changes in the central nervous system (CNS). Thus, brain manifestations like convulsive seizures, anosmia, ageusia, headache, cerebrovascular accidents, acute necrotizing hemorrhagic encephalopathy, acute encephalitis, and the virus presence in the cerebrospinal fluid are increasingly found by doctors and researchers worldwide [7–11].

These clinical findings are supported by studies on mechanisms and consequences of COVID-19-induced damage to the CNS [12]. Specifically, experiments with transgenic mice expressing human ACE2 have shown that SARS-CoV-2 enters the brain through the nasal cavity epithelium and then spreads through the brain tissue: at the first 4 days, the virus was detected in the piriform cortex, basal ganglia, midbrain, and hypothalamus; all these structures are directly or indirectly associated with the olfactory system [13]. Later (by day 4), the substantia nigra of the midbrain, amygdala, hippocampus, and cerebellum are affected; in the above experiments, this damage caused severe neurological dysfunction or death of animals. These outcomes were associated with massive neuronal death, but without signs of severe neuroinflammation, astrogliosis, or microglia activation. However, by day 4, a pronounced hyper-production of pro-inflammatory cytokines was detected in the brain tissue that, probably, contributed to the high mortality. Notably, lung infiltrates emerged after CNS dissemination with the virus, and the lung damage by itself was not the direct cause of the animals’ death [13].

**Figure 1:**
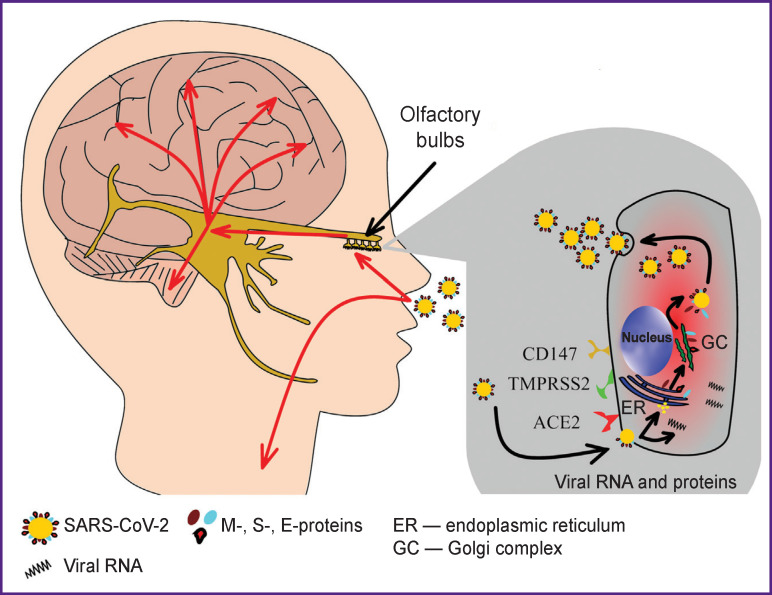
Mechanisms of coronavirus infection (designed by the authors)

The sensitivity of human brain cells to SARS-CoV-2 was confirmed experimentally *in vitro* [14]. One study analyzed the expression of ACE2 in cells of the nasal cavity. It was shown that olfactory neurons were not the cells mostly expressing ACE2 as a receptor for coronavirus; this role was attributed to epithelial cells and stem cells, especially, horizontal basal cells involved in the regeneration of the olfactory epithelium after its damage [15]. Another study suggests that respiratory failure in SARS-CoV-2 infection may have a central mechanism originated in the affected brain [16]. In [17–19], several mechanisms of the neurotropic activity of SARS-CoV-2 are systematized. The mechanisms include the virus attachment to ACE2 on endothelial cells of cerebral capillaries (in case of viremia) or on cells of the olfactory epithelium, followed by virus penetration through the palatine plate into the brain tissue in the immediate vicinity of the olfactory bulbs (Figure 1). Possible breach of the blood-brain barrier (BBB) was also discussed; the authors called for further studies on that subject, especially considering the recently reported formation of micro-hemorrhages [16] (see Figure 1).

SARS-CoV-2 contains several principal proteins [20]: M-protein (26 kDa) participates in the assembly and formation of new viral particles (budding); E-protein (9 kDa) — in the assembly and release of viral particles from the cell, it also has the properties of viroporin (an ion channel permeable to calcium, sodium, and potassium); S-protein (150 kDa) facilitates virus binding to the cellular receptor (in particular, ACE2) and entry into the cell [21]; nucleocapsid N-protein is involved in packaging of viral RNA; and ORF8b is a protein, which, being in an aggregated state, can induce stress in the endoplasmic reticulum (ER stress), damage to lysosomes and apoptosis; in addition, together with E-protein and 3a-protein, ORF8b can induce the formation of NLRP3 inflammasomes in target cells (for example, in macrophages) [22–24]. Interaction of the coronavirus S-protein with ACE2 results in a decrease in the expression of ACE2 in the lung tissue of experimental animals [25]. This result suggests that ACE2-mediated conversion of angiotensin II (AT II) to AT 1-7 and the subsequent effect of AT 1-7 on Mas receptors will be suppressed in cells infected with SARS-CoV-2. The N-protein of SARS-CoV-2 interacts not that much with CD147 but rather with cyclophilin A (CypA or peptidyl-proline isomerase) — the ligand for CD147 [6]. Moreover, the same ligand can act as a target for the entire order of nidoviruses [26]. That may explain why inhibitors of the peptidyl-proline-isomerase activity in cyclophilin A (for example, cyclosporin A) block the replication of arteriviruses and coronaviruses [27]. Some authors [26, 28] suggest that the FK506-binding protein is also involved in the complex formed between the coronavirus N-protein and cyclophilin A. Their combined activity provides, on the one hand, viral replication and, on the other hand, it controls the antiviral immune response through the NF-AT driven gene expression and the changes in the calcineurin activity in target cells. It is important to know more about the effects of SARS-CoV-2 on the neurovascular unit of the brain.

## Neurovascular unit of the brain

The structure of the neurovascular unit (NVU) of the brain incorporates endotheliocytes, pericytes, perivascular astrocytes, neurons, and, according to [29], microglia cells; all these elements interact with each other anatomically and functionally. The NVU of the brain is a dynamic system that protects the CNS from toxins, modifies the blood supply to active areas, and selectively transports metabolites and neurotransmitters from the brain to the blood [30].

In the NVU, interactions and metabolic conjugation between the cells, as well as implementation of the BBB function, are carried out by various transport and signaling mechanisms. It has been experimentally proven [31] that the structural and functional integrity of the BBB requires coordination between the astroglial molecules involved in local metabolic conjugation with endothelial cells within the NVU. These mechanisms are vulnerable to various damaging factors such as hypoxic exposure, bacterial and viral inflammation, or neurodegenerative diseases [31]. In particular, this vulnerability pertains to the systems implementing the reception and transport of lactate and NAD^+^ (monocarboxylate transporters, molecules of Cx43, CD38, and GPR81) [32, 33] as well as CD147, which is functionally conjugated with monocarboxylate transporters (MCT1, MCT4) and gamma-secretase. The latter is involved in proteolysis of the amyloid precursor protein and regulates the expression and activity of matrix metalloproteinases [34, 35].

SARS-CoV-2 is able to enter the CNS through the vascular and lymphatic systems [36]. For example, this virus can infect white blood cells and migrate with them to the brain. Viral particles could also be transported through the BBB when its permeability is impaired. Recent studies have shown that SARS-CoV-2 can cross into peripheral lymphatic vessels connected to the brain’s glymphatic system [37]. Therefore, impaired integrity of the BBB (primarily, the endothelial layer of the cerebral vessels) is a significant factor of CNS damage in COVID-19. Given that some strains of coronaviruses have a tropism for vascular endothelial cells of the mammalian brain [38], the damage to the endothelial layer of blood vessels and the increase in BBB permeability becomes substantiated even more.

Thus, the increased BBB permeability facilitated viral invasion into the brain [39]; the authors explain this observation by a sharp decrease in the expression of the tight junction proteins ZO1, VE-cadherin, and occludin, but not claudin-5 (*in vitro* and *in vivo*). According to the authors, the decreased value of trans-endothelial resistance (*in vitro*) — an overall indicator of integrity of the endothelial monolayer and the BBB — also played a role.

It is, therefore, reasonable to point to the NVU as a major target attacked by SARS-CoV-2 in the central nervous system, including damage to the endothelial cells of the cerebral vessels. In support to this mechanism, it can be noted that endothelial cells express ACE2, which plays a key role in the pathogenesis of COVID-19 [40, 41].

## ACE2 and products of its catalytic activity in the brain

For decades, AT II was considered the major final product and biologically active peptide of the renin-angiotensin system (RAS). However, recent studies demonstrated that other AT II derivatives could also have a biological activity [42]. The main peptide products in the RAS result from two enzymatic pathways: angiotensin-converting enzyme (ACE), which catalyzes the conversion of AT I to AT II, and ACE2, which catalyzes the production of other peptides.

According to various sources, ACE2 shares 42–61% homology with ACE; it exhibits the maximum enzymatic activity at pH 6.5, and it differs from ACE in the catalytic characteristics (its only active site functions as a carboxypeptidase). In addition, it is believed that ACE inhibitors do not affect the activity of ACE2 [43, 44]. Unlike ACE, ACE2 is expressed predominantly in the lungs, heart, and kidneys [45, 46], as well as in the brain tissue [43]. It is noteworthy that ACE2 is involved in the catalytic conversion of other peptides as well (e.g., neurotensin, dynorphin, and bradykinin derivatives); the biological significance of these processes is yet to be studied [47].

By interacting with AT I, ACE2 converts it into AT 1-9 and further into AT 1-7, which control the vascular tone, cell proliferation, and inflammation. By acting on AT II, ACE2 can directly convert it to AT 1-7, while the alternative pathway for AT II (via aminopeptidases A and M) leads to the formation of AT III and AT IV [48]. Proto-oncogene Mas (GPCR) and insulin-regulated aminopeptidase (IRAP) serve as receptors for AT 1-7 and AT IV, respectively [49, 50]. Some authors believe that signal transduction via the ACE2/AT  1-7/Mas system contributes to cell protection in cardiovascular diseases, renal pathology, and insulin resistance, while the AT IV/IRAP pathway may have a role in protecting brain cells from cerebral ischemia and in reducing a memory deficit (see the table).

**Table:** Physiological and pathophysiological actions of the main angiotensin II degradation products in the brain tissue

Degradation products	Action	Source
AT 1-7	Participating in the implementation of learning and memorization	[1, 51]
	Preventing the norepinephrine release, increasing the local production of bradykinin and nitric oxide Stimulation of vasopressin secretion	[43]
	Regulation of vascular tone, cell proliferation, inflammation	[48]
	Protection of NVU cells in insulin resistance, cardiovascular and renal diseases	[49, 50]
	Reducing anxiety Modifying the production of corticotropin-releasing hormone in the hypothalamus	[52]
	Decreasing the production of free radicals Initiation of redox signal transduction	[53]
	Modifying the expression of tight junction proteins (claudin-5, ZO1) Reducing the expression of matrix metalloproteinase MMP-9 Stimulation of ATP production Preventing mitochondrial fragmentation	[54, 55] [56]
AT IV	Protecting brain cells from ischemia, reducing memory deficit	[49, 50, 57, 58]
	Inhibiting the catalytic activity of IRAP Modifying the glucose transport into cells	[59]
	Inhibition of cysteine aminopeptidase (improving learning and memorization) Anticonvulsant and antiepileptic action Control over the cerebral vascular tone	[59, 60]
	Accumulation of endogenous oxytocin	[61]

In the brain tissue, ACE2 is expressed on microvascular endothelial cells [62], as well as in neuronal and astroglial cells [43]. It is believed that astrocytes produce angiotensinogen, which is converted by ACE2 in neurons into final peptides having biological activity. Notably, the activity of ACE2 in brain cells is significantly higher than that of ACE; its highest level is found in the hypothalamus and the lowest one — in the pituitary gland, with the hippocampal ACE2 activity in the middle [63].

The functional role of ACE2 in the brain is traditionally attributed to the central regulation of the cardiovascular system and water-mineral metabolism [64]. However, evidence is accumulating that the local effects of the RAS peptides in the brain may as well involve cognitive functions, behavior, etc. [65, 58]. Some authors believe that local changes in the brain RAS activity can be associated with chronic neurodegeneration (Alzheimer’s disease and Parkinson’s disease) [66] or ischemic brain damage [67]. For example, in the cerebral cortex of patients with Alzheimer’s disease, a decrease in the ACE2 activity and a negative correlation with the ACE activity was found; further, changes in the AT II/AT 1-7 ratio were not consistent [68]. There is no information on a possible role of ACE2 in the pathogenesis of cerebral amyloid angiopathy (CAA); that stands in contrast to ACE1, whose localization indirectly indicates its contribution to the pathogenesis of CAA [69]. In general, the increased ACE activity and the decreased ACE2 activity in Alzheimer’s disease correlate with the contribution of ACE and ACE2 to the proteolysis of beta-amyloid (Aβ): thus, ACE2 converts the highly amyloidogenic Aβ43 to Aβ42, which is then converted (with the help of ACE) to Aβ40, which is less capable of forming amyloid aggregates in the brain tissue [70, 71].

Overexpression of ACE2 in the brain causes the changes typical of arterial hypertension, myocardial hypertrophy, and chronic heart failure (due to the central dysregulation of the cardiovascular system and water-electrolyte metabolism). Limited brain damage during ischemia and moderate post-ischemic cognitive deficit are associated with reduced production of free radicals following the generation of AT 1-7, but not AT II; likewise, reduced anxiety was explained by the effect of AT 1-7 on the production of corticotropin-releasing hormone in the hypothalamus. Animals lacking ACE2 expression in the brain develop oxidative stress, cognitive deficits, and memory problems; they show inhibited neurogenesis in the hippocampus (especially during exercise, which should stimulate neurogenesis), and a reduced response to the vasodilating action of acetylcholine [1, 52].

It has been experimentally proven that AT 1-7 prevents the release of norepinephrine in the brain tissue, increases the local production of bradykinin and nitric oxide (which contributes to hypotension), and stimulates the central secretion of vasopressin [43]. High expression of AT 1-7 receptors was found in the hippocampus, which was considered important for the implementation of learning and memorization [1]. Interestingly, the enzyme neprilysin, an endopeptidase involved in the metabolism of beta-amyloid, and the enzyme oligopeptidase can convert AT II to AT 1-7 in the hippocampus [72]. These enzymes can provide intramitochondrial conversion of AT II to AT 1-7, as shown in renal cortical cells where mitochondria express Mas receptors [73].

Mediated by Mas receptors in brain cells, AT  1-7 affects the activity of mitochondria and thus suppresses the production of reactive oxygen species that triggers redox signal transduction [53]. It is noteworthy that the effects of AT II can be oppositely directed. An imbalance in the local production and conversion of AT II can cause either oxidative stress (the effects of AT II prevail) or inhibition of optimal redox signaling in cells (the effects of AT  1-7 prevail). In tumor cells, activation of ACE2/ AT  1-7/Mas signal transduction leads to suppression of the store-dependent calcium entry by suppressing calcium channels ORAI1 in combination with STIM1 protein, causing a decrease in the activity of NF-kB — mediated transcription mechanisms [74].

By interacting with the Mas receptor in the hippocampus, AT 1-7 can influence the memory [51]. Stimulation of ACE2 activity in the hippocampus in experimental Alzheimer’s disease reduces cognitive dysfunction, amyloid deposition, and neuroinflammation [65].

Angiotensin IV has a wide range of effects, including the ability to improve learning and memorization, protection against cerebral ischemia, regulation of cerebral vascular tone, as well as anticonvulsant and antiepileptic activities. Some of these effects are mediated by AT receptors; others, most likely, result from AT  IV binding to IRAP. In this regard, three hypotheses have been put forward: 1) AT IV inhibits the catalytic activity of IRAP; therefore, its effects *in vivo* may be due to the accumulation of neuropeptides — IRAP substrates; 2) IRAP co-localizes with the GLUT4 glucose transporter in various tissues, and therefore AT  IV can influence the glucose entry into cells; 3) a more intriguing hypothesis suggests that IRAP may function as a receptor and, hence, represent an AT  IV agonist [59]. The angiotensin system is involved in the processes of neurogenesis at the late stages of ontogenesis, and AT  IV has a protective effect on the brain in acute cerebral ischemia [57, 58]. Angiotensin IV inhibits cysteine aminopeptidase, also known as insulin-regulated aminopeptidase (or oxytocinase), and improves memory in animals. It has been shown that the procognitive effects of AT  IV can be mediated by the accumulation of endogenous oxytocin [60]. In particular, *in vitro* experiments indicate that AT IV inhibits the activity of AT IV receptor peptidase thus leading to accumulation of several neuropeptides, including oxytocin [61].

The expression of ACE2 in the microvascular endothelium of the brain indicates that the functional activity of this molecule may be associated with maintaining the integrity of the BBB. Indeed, the reduced expression of ACE2 leads to the development of endothelial dysfunction in cerebral vessels under oxidative stress and aging [70]. Interestingly, an increase in the production of AT  II in hippocampal cells induced by surgery may change the BBB permeability [75]. It is reasonable to assume that an insufficient conversion of AT II into AT 1-7 and/or AT IV (e.g., due to inhibition of ACE2) may also lead to this effect. It has been shown that AT 1-7 contributes to the maintenance of the BBB integrity in rats *in vivo*, most likely by controlling the expression of tight junction proteins (claudin-5, ZO1, and by suppressing the expression of matrix metalloproteinase MMP-9 [54, 55]. It is noteworthy that in neuronal cells, the ACE2 activity can be suppressed by an excess of glutamate (for example, during the development of excitotoxicity) [76]; therefore, neuronal damage by glutamate within the brain NVU also leads to a decrease in the total activity of ACE2 and damage to the BBB. Moreover, the activation of Mas in the cerebral endothelium prevents their damage [77], which implies the mediating role of AT 1-7 in this effect. It has been experimentally shown that ACE2 molecules can be transported (as part of exosomes) from endothelial progenitor cells to mature aortic endothelial cells, providing the latter with increased viability, restoring the AT  II/AT  1-7 balance, and improving the mitochondrial function (stimulation of ATP production and suppression of mitochondrial fragmentation) [56]. The effect of ACE2 on mitochondrial metabolism has been demonstrated in muscle cells [78]. The question remains open as to how likely and effectively such a mechanism would function in the cerebral endothelium, and whether it would be involved in regulating the transport across the BBB.

According to one report, AT 1-7 does not affect the integrity of the BBB as shown in animals with an induced stroke [79]. Until now, the role of ACE2 and the products of its catalytic activity earned little attention as far as the BBB permeability and dysfunction of the microvascular endothelium are concerned.

## CD147 and cyclophilin A in the brain

CD147 (inducer of extracellular matrix metalloproteinases — EMMPRIN basigin) is a highly glycosylated transmembrane protein that belongs to the immunoglobulin superfamily. It is associated with caveolin-2. The ligands of CD147 include cyclophilins A and B, the RH5 protein of Plasmodium falciparum (PfRh5), and integrins. The close functional association of CD147 with monocarboxylate transporters (MCTs) of lactate and pyruvate has been confirmed: CD147 supports the functioning of MCT. In addition, CD147 is essential in controlling gamma-secretase that catalyzes proteolysis of amyloid precursor protein to amyloid beta (CD147 suppresses the activity of this enzyme). The contribution of CD147 as an activator of matrix metalloproteinases has also been noted [80]. The role of CD147 in the pathogenesis of infections caused by human immunodeficiency virus, herpesvirus, and hepatitis B virus has been highlighted [81].

It is noteworthy that animals knocked out for the CD147-encoding gene exhibit pronounced neurological symptoms (difficulties in learning and memorizing; behavioral disorders) [82]. In general, however, the role of CD147 in the brain tissue is not well understood. It is known that this molecule is expressed on endothelial cells of the cerebral microvessels, as well as on leukocytes harbored in the brain tissue during neuroinflammation [83]. We have shown that a decrease in CD147 expression correlates with an activation of GPR81-receptors for lactate in endothelial cells of brain microvessels *in vitro* [34]. In a transgenic mice model of Alzheimer’s disease (5xFAD), we found a significant decrease in the expression of CD147 in the CA3 region of the hippocampus; in animals of the control group, the dentate gyrus had a lower (compared to other regions of the hippocampus) level of CD147 expression [35]. In some studies, an increase in the CD147 expression correlated with the development of Alzheimer’s disease [84–86]; in other studies, a decrease in the expression of CD147 in brain cells caused increased production of amyloid beta [87–89].

An increasing number of experimental studies confirm the significance of CD147 in maintaining the structural and functional integrity of the BBB [82], which is not surprising given the important role of matrix metalloproteinases in controlling the BBB basement membrane permeability [90, 91], and the role of MCT in the astrocyte-endothelial interactions [92].

The CD147-associated activity was found in the mitochondria of various cell types (for example, in melanoma cells) [93]; deficiency of CD147 leads to inhibition of complex I of the respiratory chain. In photoreceptor cells, underexpression of CD147 leads to disruption of the cellular microarchitecture, as manifested by the abnormal localization of the nucleus and mitochondria [93]. The above mechanisms involving the CD 147-expressing cells in the brain remain unknown. Even less is known about the role of FK506-binding protein (FKBP) in mediating the effects of CD147 in brain cells. The involvement of FKBP seems highly likely, considering, for example, the role of FKBP in the regulation of calcium release from intracellular stores to the cytosol in neurons, astrocytes, and endothelial cells (triggered by cyclic ADP-ribose binding) [94, 95].

CD147 and ACE2 were suggested to mediate the damage caused by SARS-CoV-2 to stem and progenitor cells in the lung tissue [4]. Based on this data, we propose a mechanism of reserve depletion in stem and progenitor cells in the brain neurogenic niches that leads to suppression of postnatal neurogenesis. From this point of view, the anosmia often found in patients infected with SARS-CoV-2 might arise from neuroplasticity disorders, similar to those in chronic neurodegeneration, which ultimately led to dysfunction of the olfactory epithelium. At least, the role of CD147 [96] and angiotensin II [97] in the regulation of neurogenesis has already been demonstrated; it remains to be seen how much this mechanism contributes to the neurotropic action of coronaviruses.

**Figure 2:**
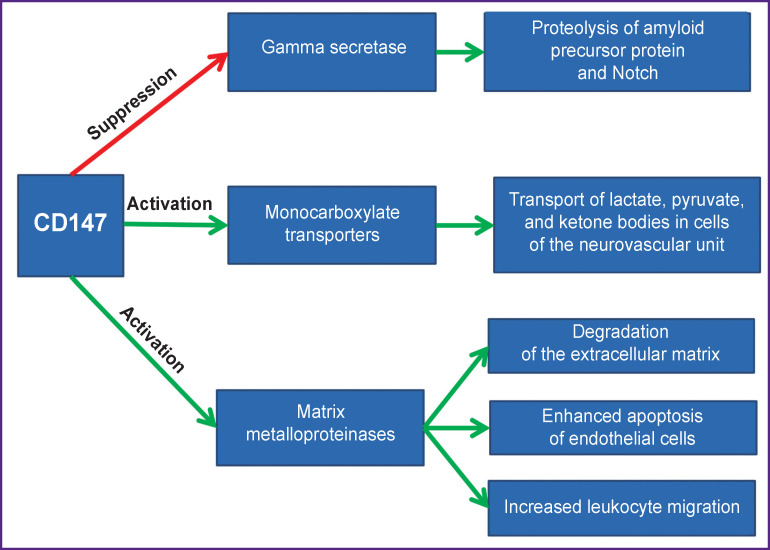
Physiological and pathophysiological significance of CD147 in microvascular cells of the brain

The natural ligand of CD147 is the extracellular cytokine — cyclophilin A. Its important role in the pathogenesis of diseases associated with oxidative stress has been shown [98]. It is not surprising that changes in the expression and activity of cyclophilin A are characteristic of chronic neurodegeneration. Specifically, apolipoprotein E of astrocytic origin causes an increase in BBB permeability associated with age and Alzheimer’s disease due to the pro-inflammatory effects of cyclophilin and the activation of matrix metalloproteinase MMP-9 [99]. Interestingly, increased levels of cyclophilin A and MMP-9 in endothelial cells and pericytes of cerebral microvessels were found in patients with Alzheimer’s disease who carry the *APOE4* gene; those correlated with BBB breakdown [100]. A study of the cerebrospinal fluid in patients with Alzheimer’s disease demonstrated that the activation of the CypA–MMP-9 mechanism is associated with pathological BBB permeability [101]. Thus, it can be assumed that a change in the nature of CypA–CD147 interactions caused by a virus leads to disruption of the structural and functional integrity of the BBB, which aggravates the neuroinflammation and interferes with the transport of endogenous and exogenous molecules into and out of the brain (Figure 2).

## Conclusion

In general, the coronavirus infection can be classified as a disease that involves damage to the neurovascular unit of the brain [102]. The pathogenesis is determined by impaired intercellular interactions (neuron-astrocytic, astrocyte-endothelial), local neuroinflammation, increased permeability of the BBB, and failed neuroplasticity; the above mechanisms include the so-called vascular component (for example, neurogenesis in neurogenic niches of the brain). However, the key questions remain unclarified: 1) why does the participation of ACE2, CD147 as targets for virus invasion into tissue lead to serious functional disorders of the CNS; and 2) is it possible to use these target molecules for pathogenetic therapy of coronavirus infection in order to protect the brain tissue? No doubt that the answers to these questions will speed up the development of highly effective treatments for coronavirus infection, including those focused on reducing the severity of neurological symptoms.
